# Genetics Reveal Long-Distance Virus Transmission Links in Pacific Salmon

**DOI:** 10.3390/ani12162120

**Published:** 2022-08-18

**Authors:** Rachel B. Breyta, William N. Batts, Gael Kurath

**Affiliations:** 1 School of Aquatic and Fishery Sciences, University of Washington, Seattle, WA 98195, USA; 2 U.S. Geological Survey, Western Fisheries Research Center, 6505 Northeast 65th St., Seattle, WA 98115, USA

**Keywords:** infectious hematopoietic necrosis virus, IHNV, steelhead, virus emergence, transmission links, Columbia river basin, full G sequence

## Abstract

**Simple Summary:**

The transmission of viruses between host populations is essential for viruses to persist on the landscape. Therefore, the identification of specific transmission links can provide insights into how a virus moves from source to recipient (sink) populations, allowing for the development of strategies to interrupt transmission routes and control viral disease. For the fish pathogen infectious hematopoietic necrosis virus (IHNV), this study identifies three transmission links associated with the emergence of IHNV in coastal Washington steelhead trout populations between 2007 and 2011. The links were identified by the genetic typing of virus isolates obtained from coastal fish and potential source fish from the Columbia River Basin. Three exact genotype matches were found, indicating at least three introductions of virus from Columbia fish to coastal fish during years of the emergence event. Likely sources were juvenile fish in the Columbia region experiencing disease, and the first detected recipient populations in all cases were adult fish returning to coastal hatcheries. Variation in timing and distance for these three transmission links will provide Pacific Northwest fish health managers with a better understanding of IHNV transmission routes from Columbia region fish to coastal steelhead trout.

**Abstract:**

In the coastal region of Washington State, a major pathogen emergence event occurred between 2007 and 2011 in which steelhead trout (*Oncorhynchus mykiss*) experienced a high incidence of infection and disease outbreaks due to the rhabdovirus infectious hematopoietic necrosis virus (IHNV). Genetic typing showed that the introduced viruses were in the steelhead-specific MD subgroup of IHNV and indicated the most likely source was a virus from the nearby Columbia River Basin. In the current study, full-length viral glycoprotein (G) gene sequences were determined for 55 IHNV isolates from both coastal and Columbia fish populations to identify specific source populations and infer mechanisms of transmission to coastal steelhead. We identified three transmission links based on exact fullG genotype matches between Columbia and coastal fish. In all cases, the likely source population was infected juvenile fish, and sink populations were adult fish returning to coastal rivers to spawn. The time intervals between detection in source and sink populations varied from 6 months to nearly 4 years, suggesting different transmission pathways. Surprisingly, distances between source and sink populations varied between 140 and 1000 km. These results confirm repeated introductions of virus from Columbia River Basin fish as the cause of emergence of MD virus on the Washington coast from 2007 to 2011.

## 1. Introduction

In the Pacific Northwest, salmonid fishes of the genus *Oncorhynchus* are hosts for the acute viral pathogen infectious hematopoietic necrosis virus (IHNV) [1]. Between the years 2007 and 2011, steelhead trout (*Oncorhynchus mykiss*) populations in many coastal rivers of Washington State suffered an exceptional period of epidemic disease events and high infection prevalence due to IHNV [2]. Although the virus was known to be endemic in the Washington coast region for several decades prior to 2007 [3], the specific subgroup of the endemic virus was UP IHNV, which has host specificity for sockeye salmon (*O. nerka*) and does not cause overt disease in steelhead [4,5]. However, the genetic typing of virus isolates from the coastal steelhead epidemics beginning in 2007 revealed the presence of a different IHNV subgroup, MD [2], which is known to have high virulence for rainbow and steelhead trout (freshwater and anadromous forms of *O. mykiss*) [5,6,7,8,9]. The finding of the MD subgroup IHNV in Washington coastal watersheds represented a major range expansion for the subgroup, which, with one exception in 1997, was previously found only in the nearby Columbia River Basin watershed that extends through much of Washington, Oregon, and Idaho [8,10].

IHNV is a global pathogen in the family *Rhabdoviridae* that was first recognized in the 1950s as the cause of a major disease, infectious hematopoietic necrosis (IHN), in sockeye salmon, steelhead/rainbow trout, and Chinook salmon (*O. tshawytscha*) [1,11]. Within these species, the juvenile age class suffers acute IHN disease with external signs of exophthalmia and hemorrhage and internal signs of necrosis in kidney and spleen; mortality can occur within days and may reach 95% depending on virus, host, and environmental conditions [12]. Adult fish generally do not suffer acute disease from IHNV infection. Both age classes shed infectious virus and IHNV transmission is primarily horizontal, through water-borne virus shed from infected fish.

At the genetic level, IHNV has diverged into several genogroups and subgroups that differ in host specificity and geographic range. In North America, IHNV occurs as three major genogroups, U, M, and L, that have specialized, with high virulence, in sockeye salmon, steelhead/rainbow trout, and Chinook salmon, respectively [10,13,14]. Relevant to the current study, the MD subgroup of the IHNV M genogroup was first detected in rainbow trout farms of southern Idaho and subsequently became established in steelhead of the extensive Columbia River Basin (hereafter referred to as the Columbia region) [7,8]. From 1994 to the present day, MD viruses have been widely distributed in the Columbia region, where they occur mostly in steelhead and cause disease epidemics in juvenile steelhead, and rarely, juvenile Chinook salmon [10,15].

A genetic typing system for IHNV field isolates was established in the 1990s based on the 303 nucleotide (nt) sequence of a variable portion of the viral glycoprotein (G) gene, called the ‘midG’ region [13,16]. This genotyping system has been used in several molecular epidemiological studies of IHNV isolates obtained from various geographic regions over the last 40 years. An early study of Washington coast isolates collected between 1976 and 2000 found only U genogroup viruses, with the single exception of one MD virus in 1997 [3]. This single early MD detection was the first emergence of MD IHNV in Washington coastal steelhead. When MD IHNV emerged in coastal Washington during the period 2007-2011, midG genotyping was used in an extensive genetic study to characterize 283 IHNV coastal isolates [2]. This typing distinguished two distinct episodes of emergence of MD group IHNV defined by different midG genotypes that were first introduced in 2007 and 2009 [2]. In combination with the earlier emergence event in 1997 [3], this was evidence of three MD emergence events that each resulted in at least one case of epidemic disease with high mortality in steelhead juveniles at coastal hatcheries. Each of the three emergence events involved a different genotype of MD group IHNV: type mG111M emerged in 1997; type mG110M emerged in 2007–2011; and type mG139M emerged in 2009–2011 [2]. Each time, the emerging genotype was widely detected in the Columbia region, at high prevalence, for at least a few years before that type emerged in the coast region [8,10]. This led to the conclusion that the most likely source of the emergent viruses in coastal steelhead was Columbia region fish populations [2]. The three coastal emergence events differed in scale, however. The first emergence of mG111M in 1997 occurred in only one coastal population of juvenile steelhead, which was destroyed when epidemic mortality became apparent. Subsequently, there was no evidence of MD virus in the coast region for a period of 10 years. The second and third emergence events, however, each occurred at several coastal sites, and despite some populations of symptomatic juveniles being culled, there was ongoing detection within the coast region for four years. Due to the widespread occurrence of dominant genotypes mG110 and mG139 in the Columbia region [10], the previous midG genotyping study was not able to resolve possible transmission sources to a finer scale.

The purpose of the current study is to provide finer resolution on possible transmission sources for the emergence events that resulted in widespread detection and several years of disease in coastal steelhead, specifically the second and third emergence events that together occurred between 2007 and 2011. To achieve finer epidemiological resolution, 55 virus isolates from the Columbia (putative source) and coast (sink) regions were analyzed here by sequencing the complete G gene, which is approximately 1500 nt in length. Since the midG region is 303 nt, this ‘fullG’ typing would provide substantially greater resolution for efforts to identify populations in the Columbia region that might have acted as sources for the coastal emergence events.

This study revolves around events initially characterized by midG typing (genotypes written as mG###M) and refined here by fullG typing (genotypes written as fG###M). These terms are easy to conflate, so a clarifying structure will be used for ease of communication: the two emergence events investigated here will be described according to their temporal incidence. We will refer to the “second emergence” as that of IHNV with genotype mG110M during the period 2007–2011, and the “third emergence” as that of IHNV with genotype mG139M during the period 2009–2011. As an ancillary goal, we also present here fullG typing of two coastal virus isolates from the second emergence period that had a variant midG genotype, mG168M. Although these isolates are not directly related to transmission links between the Columbia and coast regions, they are of interest because they are hypothesized to represent a rare example indicating virus transmission from infected juvenile hatchery fish to free-ranging fish returning to a different hatchery farther upstream [2].

## 2. Materials and Methods

### 2.1. Isolate Selection and RNA Extraction

IHNV viruses are members of the species *Novirhabdovirus salmonid*, in the genus *Novirhabdovirus*, in the family *Rhabdoviridae* [17]. A set of 55 isolates of IHNV from fish populations that were previously genotyped by midG sequence were selected for G gene sequencing. By midG sequence types, these included 35 isolates with mG110M and 18 isolates with mG139M from both coast and Columbia regions and 2 isolates with mG168 from the coast region (Table 1). RNA was extracted from isolates according to the protocol described in Breyta 2013. Briefly, viral genomic RNA was extracted from 200–500 μL of virus culture supernatant with TriReagent (Sigma-Aldrich, St. Louis, MO, USA) according to the manufacturer’s directions with tRNA (Promega Corporation, Madison, WI, USA) added to aid in RNA precipitation.

### 2.2. Sequence Analysis

Reverse transcription PCR (RT-PCR) and sequencing of overlapping cDNA fragments to determine the complete G gene nucleotide sequence was conducted as by Garver et al. [4]. Briefly, avian myeloblastosis virus (AMV) reverse transcriptase and Taq polymerase were combined with template RNA and IHNV-specific primers in one 50 μL reaction that was amplified in 30 cycles of PCR. The PCR product was purified away from amplification components using Strataprep PCR purification columns (Agilent Technologies, Santa Clara, CA, USA), and 0.5 μL was used in each of two 10 μL Big Dye Terminator (Applied Biosystems, Waltham, MA, USA) sequencing PCR reactions, using the primers previously described [4]. All PCR reactions were performed in a PTC-100 thermocycler unit (Bio-Rad, Hercules, CA, USA).

Electropherograms were inspected individually for quality, and then the three forward- and three reverse-primed sequence fragments were aligned in Sequencher v5.2.4 (www.genecodes.com (accessed on 1 September 2019)). Sequences were trimmed to the known G gene coding region of 1513 nucleotides (nt), including start methionine and stop codon and excluding the polyA tail, and exported via simple text. Every unique sequence within the dataset was given a universal sequence designator (USD), following a modification of the established midG genotyping USD nomenclature [18]. FullG sequence types here are designated as fG USDs in the format fG###M, where ### is an arbitrary three-digit designation assigned to a specific full G nt sequence, and M indicates that all isolates analyzed in this study are in the M genogroup of IHNV. This format differs from the midG sequence types of 303 nt, which are designated in the format mG###M. RNA viruses produce high numbers of variant genomes during replication, and occasionally, heterogeneity within a virus isolate is detectable as one or more bases within the sequence that produces more than one fluorescently labeled base identity. If this phenomenon is detected in the electropherograms from two or more primers, it is considered real and not an electrophoresis or software artifact. Such sites are referred to as ‘heterogeneous’, and when only one is detected within the target sequence range, that isolate is assumed to contain two discrete genomes, both of which are given fG USD type names that are shown as fG###M/fG###M*, where the asterisk indicates one site of heterogeneity. However, if more than one heterogeneous site is detected, it is not possible to determine which base identities exist together on individual genomes without subcloning, so these isolates are indicated by the fG USD type they most closely resemble followed by two or more asterisks to indicate the number of heterogeneous sites. Pairwise sequence comparisons were conducted in MEGA software v7 (www.megasoftware.net (accessed on 1 September 2019)).

### 2.3. Phylogenetic Analysis

Isolate sequences from this study were combined with other IHNV fullG sequences from North America and aligned using ClustalX (www.clustal.org, 1 September 2019) with stringent gap penalties. Phylogenetic analysis was conducted under the coalescent theory using Bayesian Markov-chain Monte-Carlo (MCMC) methods, using the BEAST software suite (v1.8.4, www.tree.bio.ed.ac.uk/software/beast/ (accessed on 1 September 2019)) [19,20]. The results were analyzed, annotated, and drawn using the complementary suite of programs, including Tracer v1.5 and FigTree v1.3.1 [21,22,23]. Dated taxa were used with a relaxed uncorrelated lognormal molecular clock prior, with a gamma-distributed rate prior extrapolated from the range of known error rates of RNA-dependent RNA polymerases [24,25,26]. The relaxed clock was used after it was confirmed that the strict clock could be rejected (95% confidence interval of the coefficient of variation and standard deviation of the relaxed clock did not span zero), and the relaxed clock yielded a positive Bayes Factor in comparison to the strict clock. The phylogenetic tree is derived from three separate MCMC analyses of 150 million generations that each achieved convergence and good mixing and were subsequently combined, summarized, and analyzed. Within the larger tree, a sub-tree represents the MD subgroup.

## 3. Results

### 3.1. Second Emergence: mG110M in the Washington Coast Region 2007–2011

**Figure 1:**
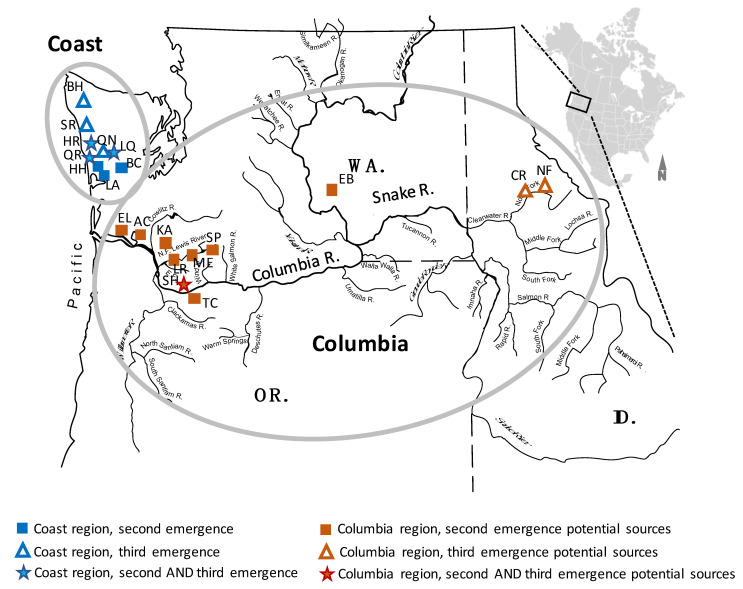
The Washington coast and Columbia regions (gray ovals) and collection sites of IHNV viruses investigated in this study. The Columbia River (mainstem and selected tributaries shown) forms part of the boundary between Washington and Oregon states, and drains all eastern rivers shown. Its largest tributary is the Snake River, which extends into Idaho. The coastal river region contains numerous smaller rivers that drain directly to the Pacific Ocean. Locations of origin for IHNV isolates in this study are indicated by symbols according to which emergence event they were related to; see legend. Blue-labeled locations are in the coastal region, while orange-labeled locations are in the Columbia region. Abbreviations for specific virus collection sites are defined in Table S1.

To identify possible transmission sources responsible for the second emergence of MD IHNV in Washington coastal steelhead, 11 isolates from the Washington coast region that were originally genotyped by midG sequencing as mG110M [2] were selected for further analysis by full G sequence (Table 1). These coastal mG110M isolates spanned all five years of the second IHNV emergence in the region from 2007 to 2011 and originated from five different sites on four different coastal watersheds (Table 2, Figure 1, Table S1). 

Nine of these isolates were from steelhead trout, one was from rainbow trout, and one was from coho salmon (*O. kisutch*). From the Columbia region, 24 isolates that were originally genotyped as mG110M [2,10] were selected for full G sequence analysis as possible sources of transmission to the coast region (Table 1). Candidate source isolates from the Columbia region were selected to represent earlier and concurrent years relative to the second emergence, from 2002 to 2011 (Table 2). The selected Columbia isolates originated from eight different sites in the lower Columbia and one site (EB) in the mid-Columbia, reflecting the general spatial distribution of mG110M isolates in the Columbia region (Figure 1, Table S1) [10]. Twenty-one of the Columbia isolates were from steelhead hosts, two were from Chinook salmon, and one was from sockeye salmon (*O. nerka*) (Table S1).

Among the 11 coastal isolates from the second emergence, four distinct fullG genotypes were found (Table 2). The earliest, genotype fG029M, was found in 2007 in a single isolate. The second genotype to appear was fG127M, which was detected in 8 of the 11 isolates and 3 of the 5 years represented, making it the dominant fullG type of the second emergence. Two additional fullG genotypes, fG137M and fG141M, appeared in one isolate each in the waning years of the second coastal emergence in 2010 and 2011 (Table 2).

Within the 24 mG110M isolates selected from the Columbia region for analysis, there were 16 unique fullG genotypes (Table 2). Of these, one was identical to the dominant fullG genotype detected in the coast, type fG127M. This type was detected in a Columbia isolate from 2004, four years before it was detected in the coast region (Table 2). This incidence of type fG127M in the Columbia region was found in October 2004 in juvenile steelhead at a facility in the lowermost tributary of the Columbia River (Figure 1, site EL). It was then detected in January of 2008 in coastal populations of adult steelhead and coho salmon at site HH, followed by detections at the same site in March–April 2008 in juvenile steelhead trout and juvenile rainbow trout experiencing epidemic disease (Table 2 and Table S1). With the exception of fG127M, the other 15 fullG genotypes found in the Columbia region were not detected in Washington coastal fish (Table 2). This lack of genotype matches with coastal isolates allows us to infer that the Columbia fish populations that were infected with those 15 fullG genotypes were not likely sources for introduction of MD virus to the coast region.

Since several closely related fullG genotypes were detected among the mG110M isolates analyzed from the coast and Columbia regions, similarity analysis was performed (Table 3). FullG nucleotide sequence types showed a greater degree of variation (up to nine nucleotide differences) than did amino acid sequences (up to four amino acid differences), which is not unexpected. However, there were a number of fullG genotypes whose nucleotide differences were silent (grey, Table 3), therefore encoding identical G proteins. For example, the first coastal MD type fG029M and the dominant coastal fG127M were detected one year apart and differ by 2 nt, but they do not differ in amino acid sequence. A similar pattern of fullG types with no amino acid differences, detected in the same years, is observed in isolates found only in the Columbia region (Table 3).

**Figure 2:**
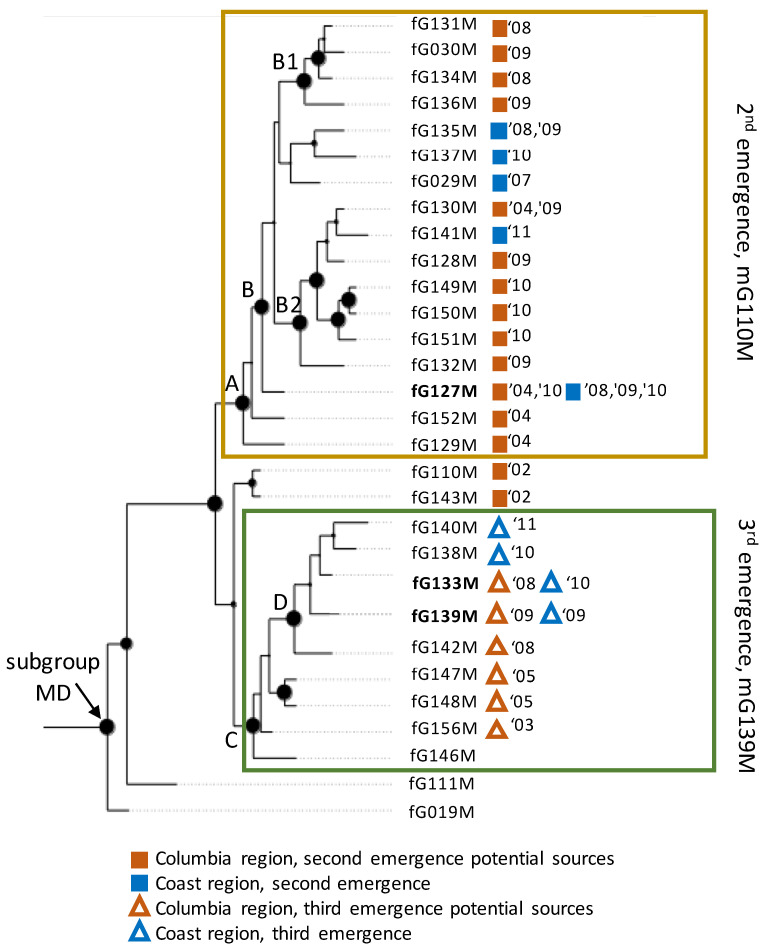
Phylogenetic tree of fullG sequence types from IHNV detections in North America—the subtree of MD subgroup fullG types is shown. Posterior probability (pp) statistics describing the likelihood that a given node existed in nature are shown as scaled circles. The fullG types originating from this study are indicated by a colored symbol (see legend), with year(s) of isolation. Letters A–D and B1, B2 indicate nodes mentioned in the text to describe the tree. The heterogeneous genotype fG129m/fG157M was not included in the analysis. FullG types found in both Columbia and coast regions are in bold text.

Phylogenetic inference was performed to provide more statistically rigorous analysis of the evolutionary relatedness of the 19 fullG genotypes found among second emergence coastal isolates and potential source isolates from the Columbia region. In the phylogenetic tree shown in Figure 2 (subgroup MD portion of the tree), the oldest Columbia types fG110M and fG143M from 2002 form a basal polytomy and the remaining 17 types form a monophyletic clade defined by node A (pp = 1.0, Figure 2). All second emergence genotypes found in the coast region were part of a monophyletic subclade defined by node B (pp = 1.0, Figure 2). Type fG127M, the only second emergence genotype to be found in both coast and Columbia regions, is basal to the rest of the types in the clade defined by node B. This indicates that there was likely one introduction event, and that other types found in the coast region evolved from type fG127M in coastal fish.

### 3.2. Third Emergence: mG139M in Coast Region 2009–2011

To investigate potential transmission sources of the third emergence of subgroup MD IHNV in coastal steelhead, ten third emergence isolates from the coast and eight candidate source isolates from the Columbia region were selected for analysis by fullG genotyping (Table 1). These isolates were all originally genotyped by midG sequencing as mG139M [2,10]. The isolates from the coast spanned all three years that comprised the third emergence event, from 2009 to 2011. They originated from adult or juvenile steelhead at six different sites in four coastal watersheds (Table 4, Figure 1, Table S1). Candidate source isolates from the Columbia region represent preceding and concurrent years from 2003 to 2009. They include three isolates from one site (SH) in the lower Columbia sub-region where mG139M virus was a non-dominant variant from 2003 to 2005, and five isolates from two sites (NF, CR) on a tributary of the Snake River in Idaho where mG139M virus was dominant from 2008–2011 [2,10,15] (Table 4, Figure 1, Table S1). The Columbia isolates were from both adult and juvenile fish, and four were from steelhead, three from Chinook salmon, and one from cutthroat trout (*O. clarkii*) (Table S1).

Among the ten coastal isolates, four distinct fullG genotypes were found (Table 4). The earliest, in 2009, was type fG139M, which was followed by types fG133M and fG138M in 2010, and type fG140M in 2011 (Table 4). Type fG133M was found in six of ten coastal isolates, making it the dominant fullG genotype of the third emergence. Variant genotype fG133M ** was also found in 2011, but it has with two sites of sequence heterogeneity and is not considered further.

Within the eight Columbia isolates, six unique fullG types were found (Table 4). These included both types fG139M and fG133M, which were also detected in the coast. Type fG139M occurred at a Snake River site in the Columbia region in 2009, the same year it was detected on the coast. Type fG133M occurred at two Snake River sites in the Columbia region in 2008, two years before it was detected in the coast (Table 4). Four of the fullG genotypes identified among potential source isolates from the Columbia region did not match any coastal genotypes. This included genotypes of all three isolates from the lower Columbia site, allowing us to rule that site out as a likely source of virus transmitted to coastal steelhead.

A pairwise comparison of nucleotide and amino acid sequences of genotypes detected in the third emergence events indicated that the two types found in both regions, types fG139M and fG133M, differ by one nt, but they do not differ in amino acid sequences. Two of the other fullG genotypes found only in the Columbia region also encode the same amino acid sequence but differ from the dominant fG133M by 1–2 nt (Table 5).

Phylogenetic analysis of the eight fullG types found in the coastal third emergence isolates and candidate source isolates from the Columbia region indicated that they are distinct from all second emergence fullG genotypes, and they are all monophyletic within a clade defined by node C (pp = 1.0, Figure 2). Furthermore, all genotypes detected within the coast region shared a monophyletic node with all genotypes from the Snake River candidate source isolates (node D, pp = 1.0, Figure 2). Within the clade defined by node D, the shared genotypes fG133M or fG139M are not shown in a basal position, but this clade has no strongly supported internal nodes so relationships within the clade are uncertain.

The finding of two fullG genotypes shared between Columbia and coastal isolates suggests that the third emergence, previously defined by midG genotyping as mG139M virus, actually involved two distinct introductions of fG139M and fG133M IHNV from the Columbia region to the coast. Both types fG133M and fG139M were detected earliest in Columbia region sites at a significantly greater distance inland than the emergent type fG127M described above. They were detected in a tributary of the Snake River, in northern Idaho state (Figure 1, sites NF and CR). Type fG133M occurred first in the Columbia region, in adult steelhead trout and Chinook salmon, as well as juvenile Chinook salmon experiencing epidemic disease, between March and August of 2008. However, this type did not appear in coastal fish until March of 2010 when it was detected in adult steelhead returning to three sites (HR, LQ, QR) on the Hoh and Quinault rivers. Additional coastal detections of fG133M then occurred between April and July 2010 in juvenile steelhead at two sites (SR and LQ) on the Salmon and Quinault rivers, and in November 2010 in adult steelhead at another Quinault River site (QN) (Table S1). The second fullG genotype fG139M was first detected in the Columbia region in the same Snake River tributary as type fG133M (site NF) but a year later, in June 2009, in juvenile steelhead trout. Only six months later was this type found in coastal adult steelhead trout returning to a site on the northern-most coastal river in our study area (site BH).

### 3.3. Coastal Transmission of Variant mG168 IHNV 2008–2009

Two coastal IHNV isolates from the second emergence time period that were originally genotyped as midG sequence mG168M were also analyzed here by full G sequence (Table 1). The mG168M genotype was shown previously to be a variant of genotype mG110M that arose in coastal fish and was detected in three fish populations during the period 2008–2009. [2]. The variant mG168M was first detected in 2008 among several minor variants in post-epidemic juvenile steelhead at coastal site LA (Figure 1). In 2009, the same mG168M genotype was found in IHNV from adult fish returning to the same site, and also adult fish returning to a different hatchery site upstream in the same watershed (site BC). This variant genotype has never been detected in any other location or time, suggesting that the viruses from 2009 were very likely to be transmission events from the first detected virus in 2008. Here, we found by full G sequencing that the original isolate from juvenile hatchery fish in 2008 and the isolate from adult fish returning to a different hatchery upstream had identical fullG genotypes, fG135M, strongly suggesting a transmission link between these coastal fish populations.

## 4. Discussion

Previous epidemiological inference of three waves of MD group IHNV emergence in the coast region of Washington State, first detected in 1997, 2007, and 2009, found evidence that the Columbia region was the most likely source in each case [2]. In the present study, higher-resolution viral genetic surveillance allowed the identification of specific fish populations with likelihood of acting as sources for the second and third emergence events that resulted in several epidemics and many detections of MD virus among coastal steelhead between 2007 and 2011. Higher-resolution genetic typing was necessary because both of the midG types associated with the overlapping 2007 and 2009 emergence events were widely distributed in the Columbia region in the years before each emergence, making it impossible to identify specific potential sources and make inferences about the transmission mechanism(s) connecting the Columbia and the coast populations.

The second coastal emergence event beginning in 2007 was originally described as being composed of viruses with the midG genotype mG110M. This type was very widely detected in the Columbia region, where it was first detected in 2002. Approximately 47 populations of fish were found with this type in the Columbia region between 2002 and 2007, with the majority in the lower Columbia sub-region. Among these, 24 representative isolates were analyzed here by fullG sequencing. The third emergence event was initially described as midG type mG139M IHNV appearing on the coast in 2009. This midG type had been detected in at least 15 different Columbia populations of fish, including those at sites in the lower Columbia sub-region where mG139 was non-dominant between 2003 and2005, and those at sites far upstream in the lower Snake sub-region where mG139M became dominant from 2008 and 2010 [2,10]. Among these, eight representative isolates were analyzed here. Thus, approximately half of the known potential source fish populations in the Columbia region were assessed here.

**Figure 3:**
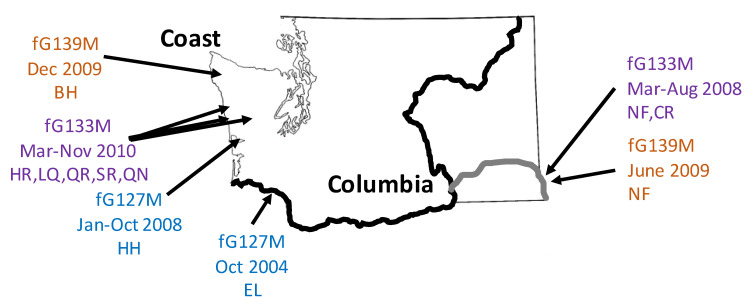
Locations and dates of detection for viruses with matching fullG genotypes identified in three transmission links of MD IHNV from Columbia region sources to coastal region steelhead sink populations.

Full G gene sequencing identified one exact sequence match, fG127M, between second emergence coastal IHNV isolates and candidate Columbia source isolates, and two exact matches, fG139M and fG133M, between third emergence coastal virus and candidate Columbia source isolates. A summary of the features of the three potential transmission links identified by matching fullG genotypes is provided in Table 6, and the spatial relationships of Columbia source and coastal sink populations are illustrated in Figure 3. As shown in Table 6, the first coastal sink populations detected for all three transmission events were adult steelhead returning to various coastal rivers. The Columbia source populations for the first two events were juvenile steelhead, and the third event had three possible source populations, including juvenile Chinook salmon experiencing an epidemic. This suggests a common pattern in which virus translocations to the coast appear to occur from juvenile fish experiencing disease in the Columbia region to returning adult fish in the coast region. In two of these events, the virus brought by infected returning adult fish to coastal hatcheries subsequently spread to juvenile fish populations at the same hatchery, causing disease, and to juvenile and/or adult fish at other hatcheries. This is consistent with conclusions from IHNV landscape transmission models that exposure to IHNV via returning adult fish is an important route of IHNV transmission to juvenile hatchery fish [27,28]. Due to this onward transmission within coastal fish populations, fG127 was the dominant fullG genotype of the second emergence, and fG133M the dominant fullG genotype of the third emergence.

Our interpretation of the matching fullG genotypes as transmission links involves some assumptions and caveats. The main assumption is that the detection of an exactly matching fullG genotype in a Columbia region fish population sampled on a date prior to the detection of the same genotype in coastal fish indicates a transmission event in which the virus from the Columbia source fish was, directly or indirectly, transmitted to coastal fish. Although the alternative explanation that the same mutation(s) could have occurred independently in both regions is theoretically possible, this is much less likely than the transmission of a pre-existing virus between fish populations, especially considering the long migrations inherent in Pacific salmonid life cycles. A caveat is that we did not sample all potential source fish populations in the Columbia region, so the same fullG genotypes identified here in transmission links may have existed in other Columbia fish populations from different locations or different years that were not tested for virus or were not selected for fullG sequencing. This could alter the temporal or spatial aspects of the transmission events described here. It is also possible that fullG genotypes in coastal fish that had no matching source could still have been introduced from Columbia fish populations that were not sampled or not sequenced. A likely example of this is coastal genotype fG029M, which was the very first MD virus detected in the second emergence in 2007, but no matching source was identified in the Columbia region. As the first detection of an MD virus in the coastal fish, it is virtually certain that this virus was introduced from outside the coast region, and phylogenetic analysis places it within the same clade as all other second-emergence Columbia and coastal isolates (Figure 2, node B). This suggests it most likely came from a Columbia region source that was not sampled. The final assumption used for interpretation of our data is that fullG genotypes that differ from sink populations can be used to “rule out” potential source populations. This was useful in providing higher resolution identification of source populations for all three transmission links identified here (Table 6).

At the landscape scale, IHNV transmission is best characterized in freshwater because of the importance of IHN disease in fish farms and conservation hatcheries that rear fish in freshwater river systems [27,28]. However, populations of fish in freshwater hatcheries are released as juveniles to migrate to the ocean, intermingling with wild fish during their natural anadromous life cycle in river, estuary, and ocean environments [10]. Therefore, virus transmission among different fish life stages or different salmonid species in these open environments is possible but generally poorly understood. Since these salmonid species eventually home to their natal freshwater sites to spawn, there are limited ways that fish originating from the different regions may interact. The three transmission links identified here can be interpreted to provide insights into the possible mechanisms of transmission of virus from the Columbia to the coast region. They differ temporally and spatially, with time intervals between source and sink detections ranging from 6 months to almost 4 years, and migration distances of 140–1000 km (Table 6). In the first transmission link, type fG127M was detected in the lower Columbia area in 2004, nearly four years before it was detected in the coast area. The source fish population in the Columbia region was juvenile steelhead trout, described as having IHN disease signs. It is possible that survivors of this disease event were released the following spring, in 2005, and retained the infection through to adulthood in the marine environment. They may have transmitted their type fG127M to adult coastal-origin steelhead trout in the marine or estuarine environments, or positive individuals may have strayed during return migration and ended up in the coastal river. Either scenario could explain the detection of adult steelhead with type fG127M in January 2008 in a coastal river. In general, steelhead trout smolts are not tagged before release like Chinook salmon, so it was not possible to determine if any of the type fG127M-positive coastal adult steelhead had actually originated in the Columbia region. As an alternative mechanism, the Columbia source fish could have transmitted fG127M virus as out-migrating smolts to other coastal smolts, and then the coastal fish retained the virus into adulthood. Either of these possible paths entails transmission of the virus between fish of similar life stages, which fits the 4-year timing interval, and both involve long-term persistence of infectious virus in some fish host. The persistence of IHNV in juveniles surviving acute infection is not well understood, and the frequency with which it may occur in hatchery fish is not known.

Whatever path of transmission followed by type fG127M, it is clear that the virus with this G gene sequence produced progeny and diversified in both source and sink regions. For coastal sink populations, this is evident as four descendant fullG types from coastal sources identified phylogenetically within the clade defined by node B (Figure 2). The presence of genotypes detected only in the Columbia region within the clade defined by this node suggests that similar evolution from type fG127M has occurred also within that region, illustrated by subclades B1 and B2. Within the sink coast populations, descendent type fG137M was detected as a variant in 2010. Genotype fG141M is also a variant of fG127M in coastal fish, but its position within the B2 subclade, distal to two strongly supported ancestral nodes that are shared only with Columbia isolates, suggests a possibility that fG141 could also represent a separate introduction to the coast of an fG127M variant that arose in the Columbia region. After 2011, neither these coastal types nor any other MD type IHNV viruses were detected in coastal fish populations. In the Columbia region, 10 fullG types descendent from fG127M were detected between 2004 and 2010 (Figure 2, subclades B1 and B2). Unlike the coast, MD group IHNV viruses continue to be detected in the Columbia region to the present day. Specifically, the midG genotype mG110M continues to be detected in IHNV positive fish populations of this region, so it is possible that either fG127M or one of its descendants is still present in the region.

FullG sequence analysis of isolates related to the third emergence event revealed three unexpected features. First, there were two unique fullG types found shared between fish populations of the Columbia and coast regions, fG139M and fG133M, implying two discrete introduction events. Second, both source populations were found deep inland, more than 640 river kilometers farther from the sink population than for the translocation of type fG127M (Figure 3). Third, there was a tight temporal association of just 6 months between the identified source Columbia population of type fG139M and the sink coast population (Table 6). The source Columbia fish population with fG139M was juvenile steelhead trout undergoing epidemic IHN disease in June 2009. These fish were not released until the spring of 2010, but type fG139M was found in December of 2009 in the sink population of adult steelhead trout returning to spawn on the north coast of Washington. It is extremely unlikely that virus shed into the hatchery effluent water in Idaho remained viable and at infectious levels all the way to the mouth of the Columbia River, where a coastal origin steelhead trout adult may have ‘dipped in’ before resuming accurate homing to spawn up the coast. Alternatively, virus shed from the identified source population in Idaho may have locally infected some unsampled population of fish, such as other out-migrating juvenile fish or steelhead kelts, that transmitted the virus to the coastal steelhead adults soon after they reached the estuary or ocean environments. Even if an unsampled fish population acted as an intermediate transmission link, fullG typing indicated that the source population for the coastal incidence of fG139M was almost certainly from the Snake River sub-region in Idaho. This is because the prevalence of mG139M in fish populations before 2008 was low, despite high levels of surveillance. There were 11 populations known to have had mG139M between 2003 and 2007, but there are 33 known during the period 2008–2012. Furthermore, all but three of these were found in the same Snake River tributary as the populations selected for analysis here, and the three from the lower Columbia sub-region were ruled out by having non-matching fullG genotypes. Regardless of the exact transmission mechanism an important inference from the short time interval between source juvenile fish in Idaho and coastal adult fish is that it must involve, at some point, virus transmission between fish of different life stages.

The temporal relationship between Columbia detection of type fG133M has an intermediate time interval with two years separating the incidence in source and sink populations (Table 6). This transmission event also involves a long-distance migration equal to that of fG139M, and it was unique in having multiple possible source and sink populations. Among three possible source populations identified within a few months of each other in 2008, two were adult fish that would not have out-migrated, so the juvenile Chinook salmon population experiencing an epidemic of fG133M in July of 2008 may be the most likely actual transmission source. Survivors of this epidemic would have been released for out-migration in 2009, so subsequent detection in adult steelhead in March of 2010 again implies transmission between different life stages of fish, likely in either estuarine or ocean environments.

The third coastal emergence event lasted from 2009 to 2011. The two emergent types fG133M and fG139M are most closely related to each other, and phylogenetic analysis revealed two fullG types that appear descendent from them in the coast region. Types fG138M and fG140M were detected in coastal fish in 2010 and 2011, and they were never detected in the Columbia region. Phylogenetic analysis did not identify fullG types that may have descended from types fG133M or fG139M after their detections in the Columbia region, however. As with the second emergence, this third emergence of MD group IHNV did not persist in the coastal region beyond 2011, despite ongoing virus surveillance.

Thus, the three transmission links identified here differed in important ways that provided novel insights. The transmission of fG127M virus involved a relatively short migration distance and a long time interval of four years, suggesting mechanisms that most likely involve transmission between similar life stages and long-term persistence of virus in either Columbia or coastal fish. In contrast, the fG139 introduction was associated with a long migration distance and a very short time interval of 6 months, suggesting the possibility of infection of an intermediate host that was not sampled here, and transmission between juvenile and adult fish. The third event, fG133M, was also a long migration distance but with an intermediate time interval of 2 years, again suggesting a mechanism involving transmission between fish at different life stages.

In addition to characterizing three transmission links associated with the second and third coastal emergence events, our ancillary goal of genotyping two coastal IHNV isolates with midG genotype mG168M identified a unique transmission link between coastal fish populations. The finding of an exact fullG genotype match greatly strengthens our previous inference that this is an example of transmission from infected juvenile hatchery fish to free-ranging adult fish [2]. This is a rare observation that is valuable in contrast to the large number of observations indicating transmission of virus to juvenile hatchery fish from free-ranging fish, typically as returning adult fish shedding virus into unsecured hatchery water supplies [15,16,27,28,29].

It is not known why the repeated introductions and emergence events of subgroup MD IHNV in coastal steelhead populations did not result in MD virus becoming endemic in the coastal region as it has done in Columbia steelhead. The regions differ in several ways that might play a role. There are more sockeye salmon populations in the coast region than in the Columbia region. Within coastal sockeye salmon, the IHNV that is most prevalent is the subgroup UP [10,30]. Although infrequent, detections of UP group IHNV have occurred in coastal steelhead trout for decades, and it has never been associated with disease signs or juvenile epidemic mortality. This may indicate that MD viruses cannot compete well against high prevalence of UP viruses. It does not appear that asymptomatic historic ‘experience’ of UP viruses has provided any form of resistance or cross-protection against MD viruses, as the mortality levels of epidemic disease during the coastal MD emergence events was quite high [2]. This high mortality phenotype was confirmed in controlled laboratory studies using several different brood years of coastal steelhead trout [5]. Another difference is that there are more hatchery steelhead trout populations in the Columbia region than on the coast. If MD group IHNV viruses needed a minimum number or density of host populations to become endemic, that threshold may not be present in the coast region. A related factor may be that the rivers in the coast region are generally considered more ‘pristine’ than those within the Columbia River Basin due to the lower human population density and fewer land-use changes that impact stream quality for salmon and trout. One interesting feature of these events is that emergence from Columbia populations occurred when MD IHNV epidemic disease frequency was high in the Columbia region. Epidemic disease in juvenile steelhead trout at hatcheries within the Columbia region is highly variable from year to year, including some years with none reported, and other years with dozens of cases [10].

Based on fullG sequence analyses presented here, the emergence of MD IHNV in coastal steelhead from 2007 to 2011 involved at least three separate introductions of virus from Columbia region sources, rather than the two introductions hypothesized from midG typing. These were identified as exact matches of fullG sequences fG127M, fG139M, and fG133M in Columbia region source fish before they were detected in coastal steelhead. In addition to the finding of exact fullG type matches, phylogenetic analyses of coastal fullG sequences also suggest the possibility of two additional translocations of unsampled Columbia MD viruses that were detected on the coast as fG029M and fG141M. Thus, the actual number of transmission events from the Columbia to coast regions is at least three, and there were possibly five introductions between 2007 and 2011. In addition to identifying more transmission links and increasing confidence in previously identified links, fullG analysis also allowed us to rule out some candidate Columbia source populations when they were found to have genotypes that did not match those in coastal fish. This was most important in the fG139M and fG133M transmission links that involved very long-distance translocation of MD virus from Idaho fish populations. In this case, there were alternative candidate source populations in the lower Columbia region that would have involved a much shorter translocation distance, but these were ruled out by showing they did not have matching fullG virus types. This allowed us to conclude with confidence that at least two transmission events involved translocations of over 900 km. Although this seems unexpected, long migrations are part of the natural life cycle of Idaho salmonid populations, and we now conclude with confidence that they can participate in transmitting virus from the Snake River region to coastal fish populations.

## 5. Conclusions

Overall, the higher resolution of full G sequencing provided stronger support for the general hypotheses derived from earlier midG typing, and it also provided novel data that advance our understanding of the mechanisms of emergence of MD IHNV in Washington coastal salmonids. We identify here at least three, and possibly five introductions of Columbia virus into coastal fish between 2007 and 2011. This suggests a continuing process of virus introduction from Columbia sources during the years of the coastal emergence, rather than one introduction that subsequently spread among coastal fish populations. The three specific transmission links identified by matching fullG genotypes varied in both timing and distance between Columbia source and coast sink populations, suggesting different transmission mechanisms. However, a common pattern in these specific transmission links is that the source populations were likely juvenile fish experiencing epidemic disease in the Columbia region, and the sink populations were returning coastal adult steelhead that carried the virus into coastal hatcheries. These repeated events occurred during a period of high MD epidemic activity in the Columbia region, and the disappearance of MD virus from the coast region after 2011 coincided with a general decline in MD IHNV detections and disease events in the Columbia region [10]. This suggests that during times of high IHNV disease impact in the Columbia region careful management of disease epidemics in Columbia hatcheries is important not only for Columbia fisheries, but for avoiding introduction of virus into coastal fish populations.

## Figures and Tables

**Table 1:** Selection of virus isolates for full G gene sequence typing to investigate emergence of subgroup MD IHNV from the Columbia River Basin in coastal Washington steelhead (*O. mykiss*). Isolates associated with the second emergence event of mG110M viruses from the period 2007–2011 and the third emergence event of mG139M viruses from the period 2009–2011 were selected for study, as well as two coastal isolates of mG168M. Available isolates and the number of fish populations they represent from the sink coast region and the source Columbia region were tallied and sub-selected for full G gene sequence typing. The number of unique fullG genotypes found in each isolate set are shown, as well as the number of types detected in both source and sink regions.

Emergence Event	Emergent midG Type	Region	# Available midG Isolates	# Positive Fish Populations	# Isolates fullG Sequenced	# Unique fullG Types	# FullG Types Found in Both
second	mG110M	Coast	44	25	11	5	1
second	mG110M	Columbia	258	47	24	16	
second	mG168 ^a^	Coast	3	3	2	1	NA
third	mG139M	Coast	203	14	10	4	2
third	mG139M	Columbia	236	15	8	6	

^a^ Coastal IHNV isolates with midG genotype mG168 are variants of mG110M that are being investigated here to test a hypothesized transmission link between coastal fish populations in the second emergence [2]. See text for explanation, NA is not applicable.

**Table 2:** Full G genotypes identified in mG110M IHNV isolates from the second MD IHNV emergence event in coastal steelhead. Regional and temporal distribution are shown for mG110M isolates in the coast region during the period 2007–2011 and for candidate source isolates from the Columbia region in preceding and concurrent years. Numbers in columns are numbers of isolates, with locations indicated as shown in Figure 1. Dashes indicate no detection of a given fG genotype in a given year.

Region	FullG Type ^a^	2002	2004	2007	2008	2009	2010	2011
Coast								
fG029M		-	-	1 SR	-	-	-	-
**fG127M**		-	-	-	6 LA,HH	1 LQ	1 HH	-
fG137M		-	-	-	-	-	1 HH	-
fG141M		-	-	-	-	-	-	1 QR
*fG135M* ^b^		-	-	-	*1 LA*	*1 BC*	-	-
Columbia								
fG110M		1 TC	-	-	-	-	-	-
fG143M		2 SH	-	-	-	-	-	-
**fG127M**		-	1 EL	-	-	-	1 KA	-
fG129M		-	3 KA,ME	-	-	-	-	-
fG129M/fG157M *		-	1 LR	-	-	-	-	-
fG130M		-	1 EB	-	-	2 KA	-	-
fG152M		-	1 ME	-	-	-	-	-
fG131M		-	-	-	1 SH	-	-	-
fG134M		-	-	-	2 SH	-	-	-
fG030M		-	-	-	-	1 AC	-	-
fG128M		-	-	-	-	1 KA	-	-
fG132M		-	-	-	-	1 ME	-	-
fG136M		-	-	-	-	1 SP	-	-
fG149M		-	-	-	-	-	1 SH	-
fG150M		-	-	-	-	-	1 SH	-
fG151M		-	-	-	-	-	2 SH	-

^a^ FullG types in bold indicate transmission link between regions. ^b^ FullG type in italics indicates two coastal isolates of midG type mG168M, a variant of mG110M, that are associated with hypothesized sink transmission [2]. FullG type fG129M/fG157M * contains one site of heterogeneity, indicated by one asterisk.

**Table 3:** Pairwise comparisons of all fullG type sequences found among second emergence mG110M isolates. Nucleotide differences are on the lower half of the matrix, and amino acids are in the upper half. Genotypes are listed according to which coast or Columbia region they were detected in, except for bold text genotypes, which occurred in both. Cells highlighted in grey indicate nucleotide differences that are silent. Genotype fG129M was excluded due to short sequence, and type fG129M/fG157M was excluded because IUPAC ambiguity codes confound pairwise analysis.

		fG029M	fG127M	fG137M	fG141M	fG110M	fG143M	fG130M	fG152M	fG131M	fG134M	fG030M	fG128M	fG132M	fG136M	fG149M	fG150M	fG151M
fG029M	Coast		0	0	2	1	1	0	1	1	1	1	1	1	1	2	3	1
**fG127M**		2		0	2	1	1	0	1	1	1	1	1	1	1	2	3	1
fG137M		3	1		2	1	1	0	1	1	1	1	1	1	1	2	3	1
fG141M		6	4	5		3	3	2	3	3	3	3	3	3	3	4	3	3
fG110M	Columbia	3	3	4	7		0	1	0	2	2	2	2	2	2	3	4	2
fG143M		4	2	3	6	1		1	0	2	2	2	2	2	2	3	4	2
fG130M		4	5	3	2	5	4		1	1	1	1	1	1	1	2	3	1
fG152M		4	4	5	8	5	4	6		2	2	2	2	2	2	3	4	2
fG131M		6	4	5	8	7	6	6	8		0	0	2	2	0	3	4	2
fG134M		5	3	4	7	6	5	5	7	1		0	2	2	0	3	4	2
fG030M		6	4	5	8	7	6	6	8	2	1		2	2	0	3	4	2
fG128M		5	3	4	3	6	5	1	7	7	6	7		2	2	3	4	2
fG132M		4	2	3	4	5	4	2	6	6	5	6	3		2	3	4	2
fG136M		4	2	3	6	5	4	4	6	2	1	2	5	4		3	4	2
fG149M		6	4	5	4	7	6	2	8	8	7	8	3	4	6		1	1
fG150M		7	5	6	3	8	7	3	9	9	8	9	4	5	7	1		2
fG151M		5	3	4	3	6	5	1	7	7	6	7	2	3	5	1	2	

**Table 4:** Full G genotypes identified in mG139M IHNV isolates from the third MD IHNV emergence event in coastal steelhead. Regional and temporal distribution are shown for mG139M isolates in the coast region during the period 2009–2011 and for candidate source isolates from the Columbia region in preceding and concurrent years. Numbers in columns are numbers of isolates, with locations indicated as shown in Figure 1. Dashes indicate no detection of a given fG genotype in a given year.

Region	FullG Type ^a^	2003	2005	2008	2009	2010	2011
Coast							
**fG139M**		-	-	-	1 BH	-	-
fG138M		-	-	-	-	1 LQ	-
**fG133M**		-	-	-	-	6 HR,LQ,QR,SR,QN	
fG140M		-	-	-	-	-	1 QN
fG133M **		-	*-*	*-*	*-*	*-*	1 LQ
Columbia							
fG156M		1 SH	-	-	-	-	-
fG147M		-	1 SH	-	-	-	-
fG148M		-	1 SH	-	-	-	-
fG142M		-	-	1 CR	-	-	-
**fG133M**		-	-	-	3 NF,CR	-	-
**fG139M**		-	-	-	1 NF	-	-

^a^ FullG types in bold indicate transmission link between regions. Two asterisks indicate one isolate contains a sequence that is partly consistent with the sequence of fullG type fG133M, but also contains two sites of heterogeneity, so proper type names are not assigned, and this genotype is not considered further (see Methods).

**Table 5:** Pairwise comparisons of all fullG types found among third (mG139M) emergence. Nucleotide differences are on the lower half of the matrix, and amino acids are in the upper half. Genotypes are listed according to which coast or Columbia region they were detected in, except for bold text genotypes, which occurred in both. Cells highlighted in grey indicate nucleotide differences that are silent. Sequences containing heterogeneous base identities are not included.

		fG139M	fG138M	fG133M	fG140M	fG156M	fG147M	fG148M	fG142M
**fG139M**	coast		1	0	1	0	0	1	1
fG138M		2		1	2	1	1	2	2
**fG133M**		1	1		1	0	0	1	1
fG140M		2	2	1		1	1	2	2
fG156M	Columbia	2	2	1	2		0	1	1
fG147M		3	3	2	3	1		1	1
fG148M		4	4	3	4	2	1		1
fG142M		3	3	2	3	3	4	5	

**Table 6:** Features of three transmission links identified from Columbia region sources to coastal steelhead sink populations.

fullG Genotype Link	fG127M	fG139M	fG133M
Coastal Emergence wave and year(s) of occurrence in Coastal region	Second emergence, dominant fG type, 2008–2010	Third emergence, first Coastal fG type, 2009	Third emergence, dominant fG type, 2010–2011
Coastal Sink Population(s): river, site, month/yr, host, life-stage(s)	Humptulips River site HH Jan. 2008 Steelhead and Coho Adult	Bogachiel River site BH Dec. 2009 Steelhead Adult	Hoh and Quinault Rivers sites HR, LQ, and QR Mar. 2010 Steelhead Adult
Columbia Source Population(s): sub-region, site, month and year host, life stage(s)	Lower Columbia sub-region site EL Oct. 2004 Steelhead Juvenile	Lower Snake sub-region site NF Jun. 2009 Steelhead Juvenile, epidemic	Lower Snake sub-region sites NF and CR, Mar.–Jul. 2008, Steelhead and Chinook Adults, Chinook juvenile, epidemic
Columbia Sources ruled out as not matching	Lower Columbia sites KA, ME, LR and mid-Columbia site EB, 2004	Lower Columbia site SH, 2003–2005	Lower Columbia site SH, 2003–2005
Time interval between source and sink detections	3 years 9 months	6 months	2 years
Approximate Distance ^a^ from source to sink site(s)	70 river km and 70 ocean km	800 river km and 200 ocean km	800 river km and 120 (LQ, QR) or 170 (HR) river km
Features of initial transmission link	Single source, 2 sink populations at site HH	Single source and sink populations	3 possible source populations, 3 sink populations
Coastal spread after first detection(s)	Spread within site HH to juvenile steelhead and rainbow trout (epidemics) Mar.–Apr. 2008, then to juvenile steelhead at sites LA and Oct. 2008 and LQ Jan. 2009	No spread detected	Spread within site LQ to juvenile steelhead July 2010, and to site SR juvenile steelhead Apr. 2010, and QN adult steelhead Nov. 2010

^a^ Distance of transmission link from source to sink detections includes river kilometers along Columbia River and tributaries in the Columbia region, and ocean kilometers northward along Washington coast from the mouth of the Columbia River. Table S1. Isolates of infectious hematopoietic necrosis virus (IHNV) from coastal Washington and Columbia River basin sites analyzed by full G sequencing.

## Data Availability

The data presented in this study are openly available in the NCBI GenBank repository as full G sequences of isolates representing each unique fullG genotype reported here, GenBank reference numbers ON924336-ON924364.

## Supplementary Materials

The following supporting information can be downloaded at: https://www.mdpi.com/article/10.3390/ani12162120/s1, Table S1: Isolates of infectious hematopoietic necrosis virus (IHNV) from coastal Washington and Columbia River basin sites analyzed by full G sequencing.
