# Supplementary material for: Genetics Reveal Long-Distance Virus Transmission Links in Pacific Salmon
Source: Animals (Basel). 2022 Aug 18;12(16):2120. doi: 10.3390/ani12162120 (PMC9405316; doi:10.3390/ani12162120)
Supplement: Supplementary file 1 [file animals-12-02120-s001.zip › animals-1780009-supplementary.pdf]

Genetics reveal long-distance virus transmission links in Pacific salmon.
Breyta, Batts, and Kurath

Table S1. Isolates of infectious hematopoietic necrosis virus (IHNV) from coastal Washington and Columbia River basin sites analyzed by full G sequencing.

Isolate Name[a]	mGUSD[b]	Region[c]	Isolation site[d]	Site code	Species[e]	Run season	Month isolated	Day isolated	Year isolated	Fish age[f]	Disease Status[g]	fGUSD[h]
**2nd emergence, candidate source isolates from the Columbia River Basin**												
TC02StA*	mG110M	CRB	Tanner Creek	TC	Steelhead	NA	5	NA	2002	Ad	dead	fG110M
SH02StJa*	mG110M	CRB	Skamania H.	SH	Steelhead	summer	7	22	2002	Juv	NA	fG143M
SH02StJb	mG110M	CRB	Skamania H.	SH	Steelhead	summer	11	7	2002	Juv	NA	fG143M
LR04StA*	mG110M	CRB	Lewis River H.	LR	Steelhead	summer	9	13	2004	Ad	NA	fG129M/ fG157M
EB04SkA*	mG110M	CRB	Eastbank H.	EB	Sockeye	NA	9	22	2004	Ad	NA	fG130M
KA04StJ*	mG110M	CRB	Kalama Falls H.	KA	Steelhead	winter	9	22	2004	Juv	NA	fG129M
KA04ChA	mG110M	CRB	Kalama Falls H.	KA	Chinook	fall	9	27	2004	Ad	NA	fG129M
EL04StJ*	mG110M	CRB	Elochoman H.	EL	Steelhead	winter	10	6	2004	Juv	NA	fG127M
ME04StAa*	mG110M	CRB	Merwin H.	ME	Steelhead	summer	12	16	2004	Ad	NA	fG152M
ME04StAb	mG110M	CRB	Merwin H.	ME	Steelhead	winter	12	22	2004	Ad	NA	fG129M
SH08StJ*	mG110M	CRB	Skamania H.	SH	Steelhead	summer	10	9	2008	Juv	NA	fG134M
SH08StAa*	mG110M	CRB	Skamania H.	SH	Steelhead	summer	11	24	2008	Ad	asymp	fG131M
SH08StAb	mG110M	CRB	Skamania H.	SH	Steelhead	winter	12	16	2008	Ad	asymp	fG134M
SP09RbJ*	mG110M	CRB	Speelyai H.	SP	Rainbow	NA	1	12	2009	Juv	NA	fG136M
AB09StA*	mG110M	CRB	Abernathy Fish Tech. Ctr.	AC	Steelhead	winter	4	27	2009	Ad	NA	fG030M
KA09StJ	mG110M	CRB	Kalama Falls H.	KA	Steelhead	winter	9	18	2009	Juv	NA	fG130M
KA09ChA*	mG110M	CRB	Kalama Falls H.	KA	Chinook	fall	9	29	2009	Ad	NA	fG128M
KA09StJ	mG110M	CRB	Kalama Falls H.	KA	Steelhead	winter	10	22	2009	Juv	NA	fG130M
ME09StA*	mG110M	CRB	Merwin H.	ME	Steelhead	summer	11	30	2009	Ad	NA	fG132M
KA10StA	mG110M	CRB	Kalama Falls H.	KA	Steelhead	winter	4	13	2010	Ad	NA	fG127M

SH10StAa*	mG110M	CRB	Skamania H.	SH	Steelhead	winter	7	26	2010	Ad	NA	fG149M
SH10StAb*	mG110M	CRB	Skamania H.	SH	Steelhead	winter	7	26	2010	Ad	NA	fG150M
SH10StJa*	mG110M	CRB	Skamania H.	SH	Steelhead	winter	8	24	2010	Juv	NA	fG151M
SH10StJb	mG110M	CRB	Skamania H.	SH	Steelhead	winter	8	24	2010	Juv	NA	fG151M
**2nd emergence, Washington Coast sink isolates**												
SR07StJ*	mG110M	Coast	Salmon River Tribal Fish H.	SR	Steelhead	NA	5	16	2007	Juv	NA	fG029M
HH08CoA	mG110M	Coast	Humptulips H.	HH	Coho	NA	1	14	2008	Ad	NA	fG127M
HH08StAa	mG110M	Coast	Humptulips H.	HH	Steelhead	winter	1	28	2008	Ad	asymp	fG127M
HH08StAb	mG110M	Coast	Humptulips H.	HH	Steelhead	winter	1	28	2008	Ad	asymp	fG127M
HH08StJ	mG110M	Coast	Humptulips H.	HH	Steelhead	winter	3	11	2008	Juv	epidemic	fG127M
HH08RbJ	mG110M	Coast	Humptulips H.	HH	Rainbow	NA	4	1	2008	Juv	epidemic	fG127M
LA08StJa	mG110M	Coast	Lake Aberdeen H.	LA	Steelhead	summer	10	9	2008	Juv	NA	fG127M
LA08StJb*	mG168M	Coast	Lake Aberdeen H.	LA	Steelhead	summer	10	9	2008	Juv	NA	fG135M
LQ09StY	mG110M	Coast	Lake Quinault Tribal Fish H.	LQ	Steelhead	NA	1	21	2009	year-ling	dead	fG127M
BC09StA	mG168M	Coast	Bingham Creek H.	BC	Steelhead	winter	3	24	2009	Ad	NA	fG135M
HH10StAa	mG110M	Coast	Humptulips H.	HH	Steelhead	summer	1	4	2010	Ad	asymp	fG127M
HH10StAb*	mG110M	Coast	Humptulips H.	HH	Steelhead	winter	1	12	2010	Ad	asymp	fG137M
QR11StA*	mG110M	Coast	Quinault R., Tahola plant	QR	Steelhead	winter	1	3	2011	Ad	asymp	fG141M
**3rd emergence, candidate source isolates from the Columbia River Basin**												
SH03CtA*	mG139M	CRB	Skamania H.	SH	Cutthroat	NA	12	16	2003	Ad	NA	fG156M
SH05StJa*	mG139M	CRB	Skamania H.	SH	Steelhead	summer	7	18	2005	Juv	NA	fG147M
SH05StJb*	mG139M	CRB	Skamania H.	SH	Steelhead	winter	7	18	2005	Juv	NA	fG148M
DW08StA*	mG139M	CRB	Dworshak National Fish H.	NF	Steelhead	winter	3	25	2008	Ad	NA	fG133M
NP08ChJ*	mG139M	CRB	Nez Perce Tribal Fish H.	CR	Chinook	spring	7	21	2008	Juv	epidemic	fG142M
NP08ChJ	mG139M	CRB	Nez Perce Tribal Fish H.	CR	Chinook	spring	7	21	2008	Juv	epidemic	fG133M
DW08ChA	mG139M	CRB	Dworshak National Fish H.	NF	Chinook	spring	8	12	2008	Ad	NA	fG133M
DW09StJ*	mG139M	CRB	Dworshak National Fish H.	NF	Steelhead	winter	6	26	2009	Juv	epidemic	fG139M

**3rd emergence, Washington Coast sink isolates**											
BH09StA	mG139M	Coast	Bogachiel H.	BH	Steelhead	winter	12	22	2009	Ad	NA	fG139M
HR10StA	mG139M	Coast	Hoh River	HR	Steelhead	winter	3	9	2010	Ad	asymp	fG133M
LQ10StA	mG139M	Coast	Lake Quinault Tribal Fish H.	LQ	Steelhead	winter	3	23	2010	Ad	NA	fG133M
QR10StA	mG139M	Coast	Quinault R., Tahola plant	QR	Steelhead	winter	3	30	2010	Ad	NA	fG133M
SR10StJ	mG139M	Coast	Salmon River Tribal Fish H.	SR	Steelhead	winter	4	14	2010	Juv	NA	fG133M
LQ10StJ*	mG139M	Coast	Lake Quinault Tribal Fish H.	LQ	Steelhead	winter	5	17	2010	Juv	epidemic	fG138M
LQ10StJ	mG139M	Coast	Lake Quinault Tribal Fish H.	LQ	Steelhead	winter	7	6	2010	Juv	asymp	fG133M
QN10StA	mG139M	Coast	Quinault National Fish H.	QN	Steelhead	winter	11	16	2010	Ad	asymp	fG133M
QN11StJ*	mG139M	Coast	Quinault National Fish H.	QN	Steelhead	winter	1	3	2011	Juv	moribund	fG140M
LQ11StA*	mG139M**	Coast	Lake Quinault Tribal Fish H.	LQ	Steelhead	winter	3	23	2011	Ad	NA	fG133M**

[a] asterisks indicate isolates with fullG sequences deposited into GenBank under the isolate name (without the asterisk), as representatives of each fGUSD sequence.
[b] mGUSD is the midG universal sequence designator used for primary IHNV genotyping, based on sequence of the 303 nt midG region in the IHNV glycoprotein gene.
[c] Region: CRB, Columbia River Basin; WA coast, Washington state coast. Double asterisk on mG139M indicates 2 sites of sequence heterogeneity.
[d] Location: H., hatchery. Full location names given here are abbreviated in the main text and figures of the paper as shown in the Site code column, with the following explanation of selected codes: Abernathy Fish Technology Center, AC; Tahola Processing plant, QR for Quinault River; Dworshak National Fish Hatchery, NF for North Fork; Nez Perce Tribal Fish Hatchery, CR for Clearwater River.
[e] Species: steelhead, steelhead trout (*Oncorhynchus mykiss*); sockeye, sockeye salmon (*O. nerka*); Chinook, Chinook salmon (*O. tshawytscha*); Rainbow, rainbow trout (*O. mykiss*); Coho, coho salmon, (*O. kisutch*); Cutthroat, cutthroat trout (*O. clarkii*).
[f] Ad, adult; Juv, juvenile
[g] asympt, asymptomatic
[h] fGUSD is the full G universal sequence designator based on the 1513 nt G gene coding sequences presented here (see methods). The USD fG129M/fG157M indicates a sequence with one nucleotide site of heterogeneity, and fG133M** indicates two nucleotide sites of heterogeneity.
NA, not applicable or information not available
